# Common variants of the *TCF7L2 *gene are associated with increased risk of type 2 diabetes mellitus in a UK-resident South Asian population

**DOI:** 10.1186/1471-2350-9-8

**Published:** 2008-02-21

**Authors:** Simon D Rees, Srikanth Bellary, Abigail C Britten, J Paul O'Hare, Sudhesh Kumar, Anthony H Barnett, M Ann Kelly

**Affiliations:** 1 Division of Medical Sciences, University of Birmingham, Birmingham, UK; 2 Heart of England NHS Foundation Trust, Birmingham, UK; 3 Warwick Medical School, University of Warwick, Coventry, UK

## Abstract

**Background:**

Recent studies have implicated variants of the transcription factor 7-like 2 (*TCF7L2*) gene in genetic susceptibility to type 2 diabetes mellitus in several different populations. The aim of this study was to determine whether variants of this gene are also risk factors for type 2 diabetes development in a UK-resident South Asian cohort of Punjabi ancestry.

**Methods:**

We genotyped four single nucleotide polymorphisms (SNPs) of *TCF7L2 *(rs7901695, rs7903146, rs11196205 and rs12255372) in 831 subjects with diabetes and 437 control subjects.

**Results:**

The minor allele of each variant was significantly associated with type 2 diabetes; the greatest risk of developing the disease was conferred by rs7903146, with an allelic odds ratio (OR) of 1.31 (95% CI: 1.11 – 1.56, *p* = 1.96 × 10^-3^). For each variant, disease risk associated with homozygosity for the minor allele was greater than that for heterozygotes, with the exception of rs12255372. To determine the effect on the observed associations of including young control subjects in our data set, we reanalysed the data using subsets of the control group defined by different minimum age thresholds. Increasing the minimum age of our control subjects resulted in a corresponding increase in OR for all variants of the gene (*p *≤ 1.04 × 10^-7^).

**Conclusion:**

Our results support recent findings that *TCF7L2 *is an important genetic risk factor for the development of type 2 diabetes in multiple ethnic groups.

## Background

The search for genes associated with type 2 diabetes mellitus has frequently been complicated by small effect sizes and poor reproducibility between studies. The recent identification of common polymorphisms within the transcription factor 7-like 2 (*TCF7L2*) gene as risk factors for type 2 diabetes, however, provides fresh hope of unravelling the genetic basis of this complex disease. The association with a microsatellite marker (*DG10S478*) in intron 3 of *TCF7L2 *was first reported by Grant *et al*. [1] in Icelandic subjects and associations with surrogate markers were subsequently confirmed in several other populations [2-11]. Polymorphisms of the *TCF7L2 *gene demonstrate the strongest and most reproducible association with type 2 diabetes of any gene reported to date; recent large-scale meta-analyses by *Florez *[12] and *Cauchi et al*. [13] reveal associations between the *TCF7L2 *SNP rs7903146 and type 2 diabetes with *p *values of < 10^-80 ^and ~10^-140 ^respectively. The precise mechanisms by which this gene affects diabetes risk are not known, but it is thought to influence insulin secretion [8,10,11,14], possibly through its role in the Wnt signalling pathway [7,15], and may also affect insulin resistance [10,11].

The South Asian population is genetically heterogeneous and is comprised of individuals originating from India, Pakistan and Bangladesh. In UK-resident South Asian populations, type 2 diabetes is around 6 times more common than in the indigenous white Caucasian population, affecting over 10% of South Asian adults [16]. Despite the high risk of the disease, few studies have attempted to characterise its genetic basis in this ethnic group. Variants of *TCF7L2 *have been studied in two South Asian cohorts to date; a UK-resident population of South Indian origin [7] and a cohort from Pune, western India [11]. The gene has not previously been investigated in any population from the north of the Indian subcontinent. In this study, therefore, we investigated the association of common polymorphisms within the *TCF7L2 *gene with type 2 diabetes in a well-characterized UK-resident South Asian population of Punjabi ancestry, originating predominantly from the Mirpur area of Azad Kashmir, Pakistan.

## Methods

Type 2 diabetic subjects (N = 831) were recruited as part of the United Kingdom Asian Diabetes Study (UKADS), a multiple risk factor intervention trial investigating the impact of a culturally-sensitive, enhanced diabetes care package on the risk of cardiovascular disease in South Asian type 2 diabetes patients living in Birmingham and Coventry, UK. All subjects were of Punjabi ancestry, confirmed over three generations, and originated predominantly from the Mirpur area of Azad Kashmir, Pakistan. Diabetes was defined using the WHO criteria [17]. Ethnically-matched normoglycaemic control subjects (N = 437) were recruited from the same geographical areas through community screening. Information concerning the relatedness between any subjects in the study was not collected. Normal glucose tolerance was defined as fasting glucose < 6 mmol/l and 2 hr glucose < 7.8 mmol/l on a 75 g oral glucose tolerance test (OGTT). Where OGTT was not feasible, normal glucose tolerance was defined as random blood glucose < 7 mmol/l. Venous blood was collected from each subject after obtaining informed consent and genomic DNA extracted using an adaptation of the Nucleon^® ^protocol (Nucleon Biosciences, Coatbridge, UK). The study was approved by the East Birmingham Research and Ethics Committee.

### SNP selection and genotyping

We genotyped four single nucleotide polymorphisms (SNPs: rs7901695, rs7903146, rs11196205 and rs12255372) that showed association with type 2 diabetes in the study by Grant *et al*. [1]. Genotyping was carried out using TaqMan SNP Genotyping Assays (Applied Biosystems, Warrington, UK) and fluorescence was measured using an ABI 7900 Sequence Detection System (Applied Biosystems). Genotyping success rate was 98.4%. Approximately 20% of the samples were re-genotyped to estimate error rate, which was zero for all SNPs.

### Statistical analyses

Genotype frequencies for each SNP were checked for Hardy-Weinberg equilibrium using a X^2 ^goodness-of-fit test. Pairwise linkage disequilibrium (LD) was estimated using Haploview version 3.2 [18]. Alleles and genotypes were tested for association with type 2 diabetes using logistic regression. Association between genotypes and continuous variables was tested using analysis of variance (ANOVA). The significance of the relationship between odds ratio (OR) and minimum age threshold was determined using linear regression. All of the above statistical analyses were implemented in SPSS version 13.0 (SPSS Inc, Chicago IL). Power calculations were performed using Excel (Microsoft^® ^Office Excel,^© ^2003).

## Results

The clinical characteristics of the subjects in our study are shown in Table 1. Age of diagnosis, high density lipoprotein (HDL) cholesterol and HbA1c data were available for subjects with diabetes only. Body mass index (BMI), waist circumference and blood pressure measurements were available for both the diabetic group and 252 subjects from the control group.

**Table 1:** Clinical characteristics of subjects studied

	Diabetic subjects	Control subjects
**n**	831	437
**Gender (M/F)**	450/381	219/218
**Age at study (years)**	56.9 ± 12.1	55.0 ± 11.8
**Age at diagnosis (years)**	49.6 ± 11.9	N/A^a^
**BMI (kg/m^2^)**	28.3 ± 4.7	28.1 ± 4.9^b^
**Waist circumference (cm)**	102.4 ± 10.7	99.8 ± 13.1^b^
**Systolic blood pressure (mm Hg)**	140.5 ± 20.9	135.7 ± 20.4^b^
**Diastolic blood pressure (mm Hg)**	84.1 ± 11.5	85.1 ± 12.1^b^
**HDL cholesterol (mmol/l)**	1.3 ± 0.5	ND^c^
**HbA1c (%)**	8.3 ± 1.9	ND^c^

Data are expressed as means ± standard deviation (SD). Mean age of control subjects at the time of the study was significantly greater than the mean age at diagnosis of the case subjects (*p *= 4.37 × 10^-13^). ^a^N/A = not applicable. ^b^BMI, waist circumference and blood pressure measurements were only available for 252 control subjects. ^c^ND = data not determined.

**Figure 1:**
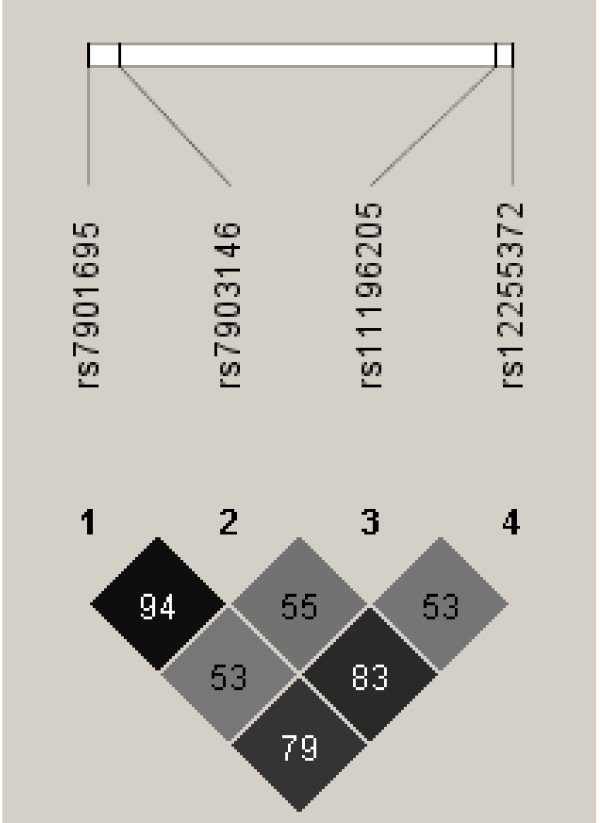
**Pairwise linkage disequilibrium (LD) between the studied variants**. LD estimated as *r*^2 ^using Haploveiw version 3.2. Strong LD is evident between all SNPs. The pattern of LD is similar to that shown in the CEPH (CEU) HapMap samples, Utah residents with ancestry from northern and western Europe, and other previously published studies involving white Caucasian cohorts.

The pattern and strength of pairwise LD between SNPs (Figure 1) was similar to that seen in the CEPH (CEU) HapMap samples, Utah residents with ancestry from northern and western Europe [19], and other previously published studies involving white Caucasian cohorts [5,9,20].

Statistical power, calculated using the number of subjects successfully genotyped, was greater than 80% for all SNPs with the exception of rs12255372. As the power of the association test for this SNP was 71.1%, we would not have been able to confidently accept the hypothesis of no association if the results had not been significant. Given the results of the present study, however, this finding is largely redundant in the context of our manuscript. It is interesting to note that, despite a smaller control group, both statistical power and the significance of the associations were greater when using a control group age cut-off of 46 years (power ≥ 94% and *p *≤ 4.90 × 10^-4 ^for all variants) compared to an age cut-off of 35 years.

### Association results

In agreement with previous studies, the minor allele of each SNP was significantly associated with type 2 diabetes (Table 2). The strongest association was seen for the variant rs7903146, with an allelic OR of 1.31 (95% CI 1.11 – 1.56, *p *= 1.96 × 10^-3^). For most variants, the risk of type 2 diabetes mellitus among minor allele homozygotes was greater than that among heterozygotes, supporting a multiplicative model of inheritance. This was not the case for rs12255372 (Table 2); however, the genotype distribution at this SNP did not conform to Hardy-Weinberg expectations in the control group (*p *= 0.02). There appeared to be an excess of minor allele homozygotes in this group, which may account for the lower than expected OR for this genotype. Genotypes of the variants studied were not significantly associated with any clinical, biochemical or morphological characteristic after correction for multiple testing. For all variants, within both the case and control groups, there was a trend suggesting that subjects with the rare allele homozygote genotypes had reduced BMI, although this did not reach statistical significance.

**Table 2:** Association of *TCF7L2 *variants with type 2 diabetes mellitus among UK-resident South Asians

SNP	Allele	Diabetic subjects F^a^	Control subjects F^a^	Genotype	Diabetic subjects n (%)^b^	Control subjects n (%)^b^	Allelic OR^c ^(95% CI)	Het OR^c,d ^(95% CI)	Hom OR^c,d ^(95% CI)
	T	0.65	0.71	TT	362 (43.7)	226 (51.7)	1.29 (1.09 – 1.54)	1.31 (1.02 – 1.68)	1.65 (1.12 – 2.44)
**rs7901695**				CT	355 (42.9)	169 (38.7)			
	C	0.35	0.29	CC	111 (13.4)	42 (9.6)	*p *= 3.60 × 10^-3^	*p *= 0.032	*p *= 0.012

	C	0.64	0.71	CC	352 (42.5)	222 (51.4)	1.31 (1.11 – 1.56)	1.37 (1.07 – 1.76)	1.66 (1.13 – 2.44)
**rs7903146**				CT	360 (43.5)	166 (38.4)			
	T	0.36	0.29	TT	116 (14.0)	44 (10.2)	*p *= 1.96 × 10^-3^	*p *= 0.014	*p *= 9.74 × 10^-3^

	G	0.53	0.59	GG	229 (27.5)	159 (36.5)	1.30 (1.11 – 1.54)	1.46 (1.12 – 1.89)	1.65 (1.18 – 2.30)
**rs11196205**				CG	417 (50.2)	199 (45.6)			
	C	0.47	0.41	CC	185 (22.3)	78 (17.9)	*p *= 1.10 × 10^-3^	*p *= 5.29 × 10^-3^	*p *= 3.33 × 10^-3^

	G	0.68	0.73	GG	382 (46.8)	241 (55.4)	1.26 (1.05 – 1.51)	1.43 (1.11 – 1.83)	1.37 (0.92 – 2.05)
**rs12255372**				GT	346 (42.3)	153 (35.2)			
	T	0.32	0.27	TT	89 (10.9)	41 (9.4)	*p *= 3.60 × 10^-3^	*p *= 5.23 × 10^-3^	*p *= 0.127

^a^F = Allele frequency. ^b^Genotype frequency expressed as number of individuals, n – values in parentheses indicate percentage. ^c^OR = odds ratio, 95% CI = 95% confidence interval. ^d^Genotype odds ratios calculated compared with homozygotes for common allele; het OR = odds ratio for heterozygous genotype, hom OR = odds ratio for minor allele homozygous genotype. All values were calculated using the full dataset, the minimum age of control subjects being 35 years.

### Effect of control group minimum age thresholds

**Figure 2:**
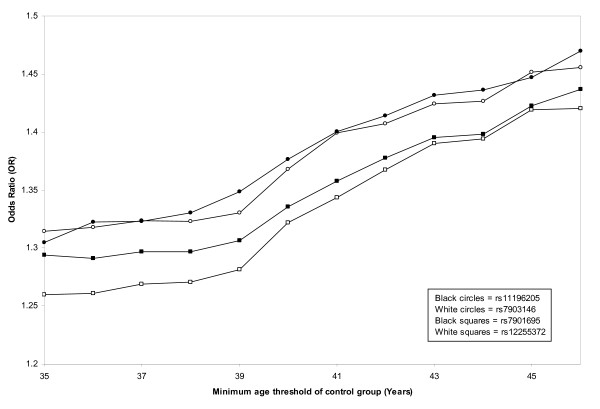
**Relationship between allelic odds ratio (OR) and control group minimum age threshold**. Allelic OR is significantly associated with minimum age threshold for all variants: rs7901695 (r^2 ^= 0.95, *p *= 8.01 × 10^-8^), rs7903146 (r^2 ^= 0.95, *p *= 1.04 × 10^-7^), rs11196205 (r^2 ^= 0.98, *p *= 1.18 × 10^-9^), rs12255372 (r^2 ^= 0.95, *p *= 4.05 × 10^-8^).

As type 2 diabetes is a relatively late-onset disease, including young individuals within the control group could potentially interfere with association tests, due to the possibility that these individuals may develop the disease later in life. One commonly used method for minimising this problem is to ensure that the mean age of the control group is at least equal to the mean age at diagnosis of subjects with diabetes. In our study cohort, the mean age of the control group was significantly greater than the mean age at diagnosis of the diabetic group (55.0 vs. 49.6 years, *p *= 4.37 × 10^-13^, Table 1), suggesting that the age of control subjects was unlikely to influence our findings. To further explore the effect of including young control subjects on the strength of association, we compared the allelic ORs calculated for each SNP using subsets of our control group defined by different minimum age thresholds. When increasing minimum age thresholds were applied, there was a corresponding increase in OR up to an age cut-off of 46 years (*p *≤ 1.04 × 10^-7^, Figure 2). When the minimum age was increased beyond this point, the relationship with OR deteriorated. It seems likely that this deterioration was due to decreasing numbers of control subjects as more individuals were dropped from the data set. The relationship between minimum age and OR was apparent for all variants, and on average the OR increased from 1.29, using an age cut-off of 35 years, to 1.45, using an age cut-off of 46 years.

## Discussion

Our results provide important confirmation that variants of the *TCF7L2 *gene are strongly associated with type 2 diabetes in populations of South Asian origin, with similar effect sizes to those seen in white Caucasian populations. Two of the variants (rs7903146 and rs12255372) have been studied previously in South Asian cohorts [7,11]. Allele frequencies for rs7903146 in our Punjabi population were similar to those reported in these studies, whereas the minor allele of rs12255372 appeared to be slightly more common in our cohort compared with these Indian populations.

Our data support the multiplicative model of inheritance for the *TCF7L2 *SNPs proposed by Grant *et al*. [1], except for the rs12255372 variant. This may be due to the minor departure from Hardy-Weinberg equilibrium observed in the control group for this SNP. All samples were re-genotyped for rs12255372 using the same technique but with fresh reagents. To rule out the possibility that the observed excess of minor allele homozygotes in the control group was due to systematical genotyping error, all subjects in this group previously genotyped as *TT *were genotyped by RFLP, using primers designed with the web-based tool SNP Cutter [21] and the enzyme Tsp509 (details available upon request). The rationale behind this latter method was that any individual falsely genotyped as *TT *by the TaqMan method would be genotyped as *CT *or *CC *by the RFLP method. Neither method revealed any inconsistencies with the initial genotyping. It seems likely, therefore, that the minor departure from Hardy-Weinberg equilibrium is purely due to chance sampling. It is worth noting that Humphries *et al*. [7] found no statistically significant evidence for a multiplicative model of inheritance for this SNP in a South Indian population.

A recent paper [22] has shown that a *TCF7L2 *haplotype (HapB_T2D_), characterised by the presence of the rs7903146 T allele, is associated with decreased BMI in subjects with type 2 diabetes. In this study we observed a similar trend, replicated for the minor allele of all variants, although this did not reach statistical significance. Unlike Helgason *et al*., however, we observed this trend within both the case and control groups. More data will be needed to investigate this relationship further.

Data concerning the relatedness between subjects in the cohort were not available in this study. It is therefore possible that some individuals are genetically related to others in the cohort, leading to non-independence of genotypes. Such cryptic relatedness potentially increases the likelihood of obtaining a false positive result in tests of genetic association, and as such must be considered as a possible limitation of our study. Given the documented reproducibility of the association between *TCF7L2 *and type 2 diabetes, however, we do not believe that cryptic relatedness has affected the results presented in this manuscript.

Our study highlights the importance of implementing minimum age thresholds when selecting control subjects for genetic studies of type 2 diabetes. Not implementing these thresholds may produce an artificially low OR, as young individuals in the control group may develop type 2 diabetes later in life and are therefore not true controls. In our study this resulted in reduced statistical power (increasing the likelihood of false negative results), a factor which could become increasingly important when studying risk factors with smaller effect sizes. The effect that a control group minimum age threshold has on an association test will depend on the prevalence of disease in the studied population. The marked effect reported in this manuscript is probably due to the relatively high prevalence of type 2 diabetes in UK-resident South Asian populations, as young control subjects have a greater chance of later developing the disease. In other populations, such as those of white Caucasian origin, these thresholds may have a smaller effect.

## Conclusion

In conclusion, our results add to the rapidly expanding body of evidence that implicates *TCF7L2 *as an important risk factor for type 2 diabetes in multiple ethnic groups.

## Competing interests

Prof. A. H. Barnett has received honoraria and fees for advisory work, together with research funding from all the companies acknowledged in the relevant section of this manuscript. He and his family hold no stocks or shares in any of the companies. None of the other authors declare any competing interests.

## Authors' contributions

SDR participated in the genotyping of samples and drafting of the manuscript, and performed statistical analysis. SB conceived of the study and participated in the genotyping and drafting of the manuscript. ACB performed DNA extraction. MAK participated in the drafting of the manuscript. All authors contributed to the intellectual content of the paper, and have read and approved the final manuscript.

## Pre-publication history

The pre-publication history for this paper can be accessed here:

http://www.biomedcentral.com/1471-2350/9/8/prepub
